# Combined Surgical Techniques for the Management of Malignant Glaucoma

**DOI:** 10.1155/2018/9189585

**Published:** 2018-11-22

**Authors:** Jinfei Tang, Ergang Du, Xingyu Li

**Affiliations:** Department of Ophthalmology, The First Affiliated Hospital of Zhejiang Chinese Medical University, 54 Youdian Road, Hangzhou, Zhejiang 310006, China

## Abstract

**Purpose:**

To characterize new combined surgical techniques for the management of malignant glaucoma.

**Methods:**

In a retrospective, interventional case series, goniosynechialysis, peripheral iridectomy, zonulo-hyaloidectomy, and anterior vitrectomy, with or without peripheral capsulectomy, were performed on nine eyes. If the patient was phakic, we performed both phacoemulsification and intraocular lens implantation.

**Results:**

Resolution of malignant glaucoma was achieved in all cases with anterior chamber deepening. Topical antiglaucoma medications were used to control the intraocular pressure in one eye. No recurrence was observed after a median follow-up of 9 months. No complications occurred during surgery or the postoperative period.

**Conclusions:**

The combined surgical methods can completely eliminate blockade and aqueous misdirection and represent a promising treatment for malignant glaucoma.

## 1. Introduction

Malignant glaucoma, known as ciliary block glaucoma or aqueous misdirection syndrome, was first described by Von Graefe in 1869. It is an uncommon and difficult-to-manage disease. Current clinical management techniques include topical cycloplegics/mydriatics, antiglaucoma medications, oral or intravenous carbonic anhydrase inhibitors, oral or intravenous hyperosmotic agents, and laser or surgical interventions [1–3]. Laser treatments include neodymium-doped yttrium-aluminum-garnet (Nd:YAG) laser hyaloidotomy/capsulotomy, argon laser treatment of ciliary processes, or transscleral cyclophotocoagulation [4–7]. Surgical interventions include conventional vitrectomy, anterior vitrectomy with posterior capsule breached, pars plana tube insertion with vitrectomy, anterior vitrectomy with iridectomy-zonulectomy, and full vitrectomy with iridectomy-zonulectomy (phacoemulsification if phakic) [8–10].

Many ophthalmologists agree that creating a passage by iridectomy, zonulo-hyaloidectomy, and vitrectomy is the most effective treatment for pseudophakic malignant glaucoma. Therefore, our goal was to characterize a simple and safe surgical procedure which limits recurrence of malignant glaucoma. Goniosynechialysis, peripheral iridectomy, zonulo-hyaloidectomy, and anterior vitrectomy, with or without peripheral capsulectomy, were trialed. In phakic patients, we performed both phacoemulsification and intraocular lens implantation in the same surgery.

## 2. Patients and Methods

Six patients (four females, two males, mean age 63 years) with malignant glaucoma in a total of nine eyes were referred to the Department of Ophthalmology of the First Affiliated Hospital of Zhejiang Chinese Medical University between December 2016 and September 2017. Malignant glaucoma was diagnosed based on a shallow or flat anterior chamber, an elevated intraocular pressure (IOP), a patent iridotomy or iridectomy or clinical slit-lamp examination or (and) ultrasound biomicroscopy (UBM) examination excluding pupillary block, and in the absence of choroidal effusion or suprachoroidal hemorrhage [11] that was unresponsive or aggravated by myotics but usually improved by cycloplegic therapy.

Preoperative assessments included previous ocular history, medical history, measurement of best-corrected visual acuity (BCVA), IOP, axial length and anterior chamber depth, and examination of the anterior segment using a slit-lamp, posterior segment fundus examination, UBM examination, and B-scan ultrasonography examination. After informed consent was obtained, all patients underwent the novel surgical management strategies described below. Patients were examined on postoperative day one, week one, month one, and then at various time intervals. The main outcome measures included anterior chamber depth, IOP, BCVA, medications, complications, and relapse (Table 1).

## 3. Surgical Procedure

**Figure 1:**
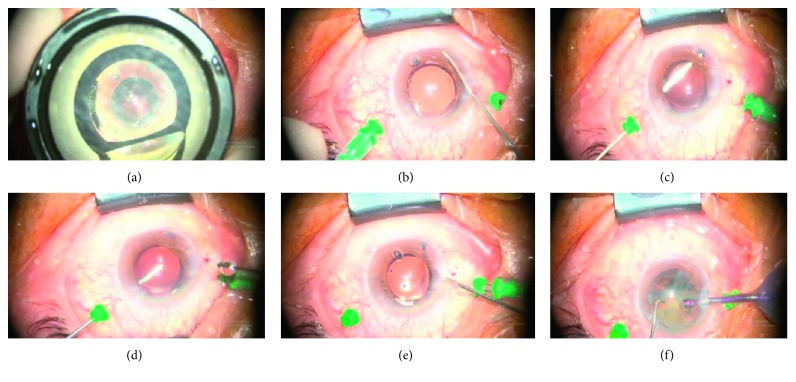
Photographs of our surgical procedure: (a) goniosynechialysis under direct visualization by the intraoperative gonioscopy. (b) Peripheral iridectomy via the anterior chamber using a vitreous cutter. (c) Zonulo-hyaloidectomy under direct vision facilitated by the enlargement of the peripheral iris opening. (d) Anterior vitrectomy. (e) Anterior chamber liquid and viscoelastic drainage and observation of rapid deepening of the anterior chamber. (f) Phacoemulsification and intraocular lens implantation if the patient was initially phakic.

All surgeries were performed by the same ophthalmologist. After postbulbar anesthesia, a 1.0 mm clear corneal incision was made at 11 o'clock. For direct intraoperative visualization using gonioscopy, a viscoelastic was used to displace the peripheral iris until the angle was opened. Two 23-gauge (G) pars plana cannulas were placed at the 2 o'clock and 10 o'clock position. One was connected to an infusion bottle of balanced salt solution at a height of 30–40 cm above the eye. A vitrector was used to make an opening in the peripheral iris through the anterior chamber. The opening was large enough to see the vitrector clearly when the vitreous cutter was inserted into the vitreous through the other cannula. Under the direct vision of the iris opening, the vitreous cutter was used to cut the lens zonules (with or without the periphery of the capsular bag), the anterior hyaloid face, and anterior vitreous. The surgery was considered successful if after the anterior chamber liquid and viscoelastic was drained, the anterior chamber rapidly deepened, suggesting that the blockade and aqueous misdirection were eliminated completely. If the patient was phakic, phacoemulsification and intraocular lens implantation was performed first (Figure 1). The operations can be combined freely. If the patients had undergone goniosynechialysis and peripheral iridectomy, they only needed the remaining operations.

## 4. Results

The patients' demographic and clinical parameters are summarized in Table 1. Malignant glaucoma occurred after phacoemulsification and intraocular lens implantation in five eyes, after trabeculectomy in one eye, after phacotrabeculectomy in one eye, and in two eyes with no surgical history. Malignant glaucoma was resolved successfully in all nine eyes by surgery with anterior chamber deepening and IOP lowering. Only one eye needed topical antiglaucoma medications to control the IOP. No perioperative complications occurred, such as iris or the trabecular meshwork hemorrhage, transient serous choroidal detachment, suprachoroidal hemorrhage, or transient exudative retinal detachment. No postoperative complications occurred, such as a transient hypotony, elevated IOP, corneal edema, fibrotic exudation, iris posterior synechia, endophthalmitis, or macular edema. No recurrence was observed after a median follow-up of nine months (range, 3–12 months).

## 5. Case (Patient 2)

A 42-year-old woman was referred to the Department of Ophthalmology, The First Affiliated Hospital of Zhejiang Chinese Medical University, in August 2017. Her chief complaint was that she had pain and soreness of the left eye for one year, which was exacerbated for one month.

**Figure 2:**
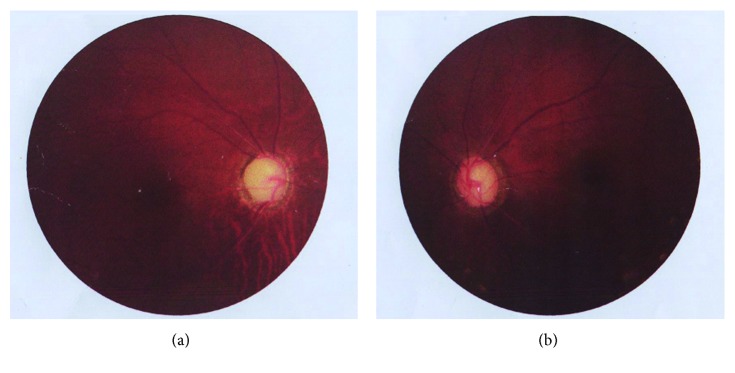
Fundus photography showing the cup/disc was about 1.0 of the right eye (a) and 0.9 of the left eye (b).

Previous to our referral, she had been treated at another hospital for the same symptoms. Ocular examination revealed that the BCVA was light perception of her right eye and 20/40 of her left eye. Both eyes showed a shallow anterior chamber and an iris laser hole at 7 o'clock in her right eye. The natural lenses were a little opaque. The cup/disc was about 1.0 of her right eye and 0.9 of her left eye (Figure 2). The IOP was 48 mmHg in her right eye and 23 mmHg in her left eye after receiving two topical antiglaucoma medications (carteolol hydrochloride eye drops twice a day and brinzolamide eye drops twice a day). Gonioscopy showed a 360-degree closed angle in both eyes. The diagnosis of primary angle closure glaucoma was considered, and trabeculectomy and iridectomy were performed on her left eye. On the first postoperative day, the IOP was 12 mmHg in her left eye. However, on the second postoperative day, the IOP increased to 22 mmHg. The patient was treated with 1% atropine ointment twice a day and carteolol hydrochloride eye drops twice a day, but the IOP increased and the anterior chamber became shallower. Because a patent iridectomy was present and no sign of choroidal effusion or suprachoroidal hemorrhage were detected, malignant glaucoma was diagnosed. A partial vitrectomy was then performed on the left eye. She was discharged from the hospital with 18 mmHg in her left eye using 1% atropine ointment twice a day and 44 mmHg in her right eye using three topical antiglaucoma medications (travoprost eye drops once a day, carteolol hydrochloride eye drops twice a day, and brinzolamide eye drops twice a day).

**Figure 3:**
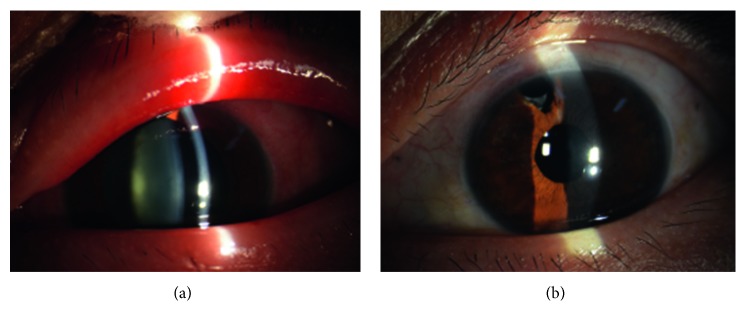
(a) Slit-lamp examination showing the left eyelid was swollen and hyperemic, with mixed congestion of the conjunctiva with a shallow anterior chamber and light cataract. The pupil diameter was approximately 7 mm before surgery. (b) Slit-lamp image showing the reduced eyelid congestion and edema, reduced conjunctiva congestion, and pupil diameter of 3 mm after surgery.

**Figure 4:**
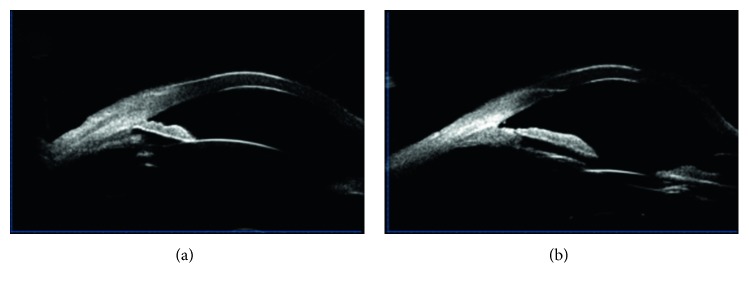
(a) Ultrasound biomicroscopy demonstrating peripheral anterior synechiae to the trabecular meshwork and a flat iris contour without pupillary block. (b) Ultrasound biomicroscopy demonstrating separation of the peripheral anterior synechiae and widening of the anterior chamber angle. The anterior chamber became deeper.

One month prior to arrival at our hospital, the malignant glaucoma in her left eye relapsed. The IOP was 38 mmHg in her left eye after application of 1% atropine ointment twice a day and four antiglaucoma medications (travoprost eye drops once a day, carteolol hydrochloride eye drops twice a day, brinzolamide eye drops twice a day, and methazolamide tablets one tablet twice daily). Furthermore, she was allergic to atropine. The left eyelid was itchy, swollen, and hyperemic (Figure 3(a)). She came to our hospital for further treatment. UBM examination showed a peripheral anterior synechiae to the trabecular meshwork and a flat iris contour without pupillary block; the central anterior chamber depth was 2.30 mm (Figure 4(a)). The patient underwent phacoemulsification, intraocular lens implantation, goniosynechialysis; and we enlarged the peripheral iris opening, and under direct visualization of the opening, zonulo-hyaloidectomy and anterior vitrectomy was performed. During surgery, an increase in the depth of the anterior chamber was readily observed. We drained the anterior chamber liquid and viscoelastic, and the anterior chamber rapidly deepened, suggesting that the surgery was successful.

On the first postoperative day, the anterior chamber remained deep and the patient's IOP measured 13 mmHg. The patient was discharged with no medications. Three months after surgery, the eyelid congestion and edema disappeared (Figure 3(b)). UBM showed the separation of the peripheral anterior synechiae and widening of the anterior chamber angle. The anterior chamber depth increased to 2.92 mm at the center (Figure 4(b)). The BCVA improved to 20/25.

**Figure 5:**
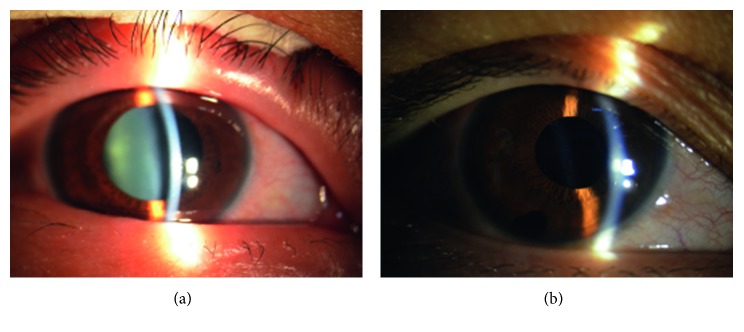
(a) Slit-lamp examination showing a shallow anterior chamber and light cataract of the right eye prior to surgery. (b) Slit-lamp image showing a deep postoperative anterior chamber. The congestion of the eyelid and conjunctiva was improved.

The IOP of the right eye was not well controlled, and the medical expenses were high. The same surgery performed on the left eye was also performed on the right eye. Subsequently, the IOP decreased to 20 mmHg with no medications and the BCVA improved to hand movement. There was no relapse during the follow-up period (Figure 5).

## 6. Discussion

The precise mechanisms of malignant glaucoma are not completely understood. Major pathophysiological factors include abnormal anatomic relationships between ciliary processes, the crystalline or intraocular lens, and the anterior vitreous face [3, 12, 13], abnormal permeability of the anterior hyaloids [3, 7], expansion of choroid [14], or anterior rotation of the ciliary body [15]. The outflow pathway of the aqueous produced by the ciliary body is blocked causing accumulation in the posterior segment resulting in anterior displacement of the iris-lens diaphragm, anterior chamber flattening, secondary angle closure, and elevated IOP. The aims of malignant glaucoma interventions are to disrupt the misdirection and restore normal aqueous flow.

The efficacy of medical treatment for malignant glaucoma is related to posterior movement of the lens-iris diaphragm and reduction of aqueous production and vitreous volume. Only about 50% of patients responded to medical therapy and recurrence of malignant glaucoma occurred when medications are stopped [12]. Laser abolition of several ciliary processes can reverse the posterior secretion of aqueous and restore the anterior chamber [5]. Nd:YAG posterior capsulotomy can reestablish forward flow of posteriorly misdirected aqueous into the drainage angle of the anterior chamber [6]. Transscleral cyclophotocoagulation may be a good option for treatment of malignant glaucoma with extensive conjunctival scarring or eyes with hazy corneas since it disrupts the ciliary-hyaloid interface and decreases aqueous production [16]. Vitrectomy can drain the aqueous outflow into the anterior chamber [17], but removing only the central vitreous is not enough, because the ciliolenticular blockade is not completely eliminated, and the aqueous may continue to accumulate in the posterior segment. The relationship between the vitreous and ciliary body that initially caused malignant glaucoma may be a key feature suggesting that the posterior capsule must be breached and the anterior vitreous removed [18]. However, relapse may occur due to connection of the periphery of the posterior capsule to the intraocular lens. Creating a permanent passage between the anterior chamber and vitreous cavity is the best way to treat malignant glaucoma. This technique eliminates aqueous misdirection and disrupts ciliolenticular aqueous blockade. The relapse rate was 66% after an anterior vitrectomy combined with an iridectomy-zonulectomy, while no recurrence occurred after a full vitrectomy combined with an iridectomy-zonulectomy within the follow-up period [9]. Incomplete removal of the vitreous may allow the remaining vitreous to move forward and block the constructed passage, promoting relapse of malignant glaucoma. Contrary to these findings, in our procedure, we discharged the anterior chamber liquid repeatedly until the anterior chamber became rapidly deepened, indicating that enough vitreous was removed and that the residual vitreous was stable and did not move forward. Total vitrectomy is probably not an option since it is difficult, time consuming, and prone to complications [19]. A similar surgical procedure in the management of pseudophakic malignant glaucoma was performed using a vitreous cutter for zonulo-hyaloido-vitrectomy through a preexisting iridectomy or iridotomy [20, 21]. In our opinion, the field of vision of this surgical procedure is limited, surgical resection is insufficient, and the residual vitreous may block the channel, promoting relapse. Anterior chamber infusion is inappropriate for malignant glaucoma surgery because it does not correspond to the normal path of aqueous humor circulation. Furthermore, anterior infusion deepens the anterior chamber artificially, leading to the premature appearance of a successful operation.

In addition, most eyes with malignant glaucoma have angle closure or angle adhesion, which can be reopened by timely goniosynechialysis. Goniosynechialysis is a surgical procedure which strips peripheral anterior synechiae from the angle wall to restore trabecular function. It can reestablish aqueous outflow through the trabecular meshwork, reducing IOP.

In the current study, the adhesive angle was separated by intraoperative gonioscopy, removal of the anterior vitreous, anterior hyaloid, and zonules (with or without the periphery of the capsular bag) facilitated by enhanced vision via the iris opening, so that in the end we were sure the channel was large enough and that we excised as much of the vitreous as possible, verifying that the surgery was successful. In addition, we used a 23G vitrector that is less traumatic, causes less conjunctival disturbance, and thus prompted faster wound healing than traditional techniques.

In conclusion, goniosynechialysis, peripheral iridectomy, zonulo-hyaloidectomy, and anterior vitrectomy, with or without peripheral capsulectomy (phacoemulsification and intraocular lens implantation if phakic) is a valuable option for the management of malignant glaucoma. The procedures can be randomly combined. Success of the surgery is determined by the direction of the aqueous flow which is more meaningful than a sudden iris movement and deepening of the anterior chamber. However, more cases, a longer follow-up period, and randomized controlled trials are still needed.

## Figures and Tables

**Table 1:** Preoperative and postoperative summary of the 9 eyes.

Patient number	Age (year)	Sex	Eye	Axial length (mm)	History	BCVA		IOP (mmHg)		Anterior chamber depth (mm)		Medications		Follow-up (months)	Complications	Recurrence
						Pre-op	Post-op	Pre-op	Post-op	Pre-op	Post-op	Pre-op	Post-op			
1	82	M	R	20.41	LPI, P	20/60	20/60	28	17	2.14	3.05	a, b	0	12	No	No
	82	M	L	20.50	LPI	20/200	20/40	18	13	1.55	3.16	0	0	12	No	No
2	42	F	L	21.71	PACG, T	20/40	20/25	38	13	2.30	2.92	a, b, c	0	9	No	No
	42	F	R	21.82	PACG	LP	HM	43	20	2.00	2.80	b, c	0	9	No	No
3	71	F	L	20.58	PACG, P, T	20/80	20/80	21	17	2.03	3.07	a, b	0	10	No	No
	71	F	R	20.55	PACG, P	20/40	20/30	23	12	1.50	3.21	a, b	0	10	No	No
4	60	M	R	22.87	P	20/200	20/200	31	22	2.30	2.86	a, b	b	3	No	No
5	59	F	L	21.49	P	20/200	20/30	46	18	1.68	3.20	a, b	0	3	No	No
6	65	F	R	22.44	Narrow angle, LPI, P	20/30	20/30	25	15	1.83	2.94	a	0	6	No	No

M, male; F, female; P, phaco; T, trabeculectomy; PACG, primary angle closure glaucoma; R, right; L, left; LPI, laser peripheral iridotomy; HM, hand movement; LP, light perception. a = mydriatics and cyloplegics; b = topical antiglaucoma medications; c = oral carbonic anhydrase inhibitors.

## Data Availability

The datasets generated and analyzed during the current study are available from the corresponding author on reasonable request.
